# Molecular Characterization of Extended-Spectrum β-Lactamase-Producing Multidrug Resistant *Escherichia coli* From Swine in Northwest China

**DOI:** 10.3389/fmicb.2018.01756

**Published:** 2018-08-03

**Authors:** Xiaoqiang Liu, Haixia Liu, Le Wang, Qian Peng, Yinqian Li, Hongchao Zhou, Qinfan Li

**Affiliations:** Department of Basic Veterinary, College of Veterinary Medicine, Northwest A&F University, Yangling, China

**Keywords:** *Escherichia coli*, antibiotic resistance, β-lactamase, OXA-48, PMQR

## Abstract

**Objectives:** The aim of the present study was to explore the prevalence and molecular characterization of extended-spectrum β-lactamase (ESBL)-producing *Escherichia coli* collected from pig farms in Northwest China.

**Methods:** Between May 2015 and June 2017, a total of 456 *E. coli* isolates were collected from fecal samples of healthy and diarrheal pigs in Northwest China to screen the ESBL producers. The β-lactamases, plasmid-mediated quinolone resistance (PMQR) genes and virulence genes among ESBL producers were corroborated by PCR and sequencing. Finally, ESBL producers were further grouped according to phylogenetic background and genetic relatedness.

**Results:** Forty-four (9.6%) out of the 456 *E. coli* isolates were identified as ESBL-producing isolates. All ESBL producers exhibited multidrug resistance (MDR) phenotype, and more than 90% of the ESBL producers were resistant to amoxicillin, amoxicillin-clavulanic acid, oxytetracycline, enrofloxacin and sulfamethoxazole/trimethoprim. All ESBL producers harbored at least one type of β-lactamase, with *bla*_CTX−M_, *bla*_TEM_, *bla*_SHV_, *bla*_OXA−48_, and *bla*_KPC−2_ being detected in forty, thirty, seven, four, two and one isolates, respectively. Sequencing revealed the most common *bla*_CTX−M_ subtype was *bla*_CTX−M−14_ (*n* = 24), followed by *bla*_CTX−M−15_ (*n* = 14), *bla*_CTX−M−64_ (*n* = 11), *bla*_CTX−M−9_ (*n* = 10) and *bla*_CTX−M−123_ (*n* = 9). *qnrS* (*n* = 23) was the predominant PMQR gene, and all PMQR genes were detected in co-existence with β-lactamase genes. *estA* (*n* = 18) and F4 (*n* = 18) were the most prevalent enterotoxin and fimbrial adhesin, respectively, and 27 different virotypes were found with respect to the association of enterotoxins and fimbrial adhesins. Twenty-four different sequence types (STs) were identified among 44 ESBL producers, and clones ST405, ST10 and ST648 were strongly present in more than one-third (34.1%) of ESBL producers.

**Conclusion:** All ESBL-producing *E. coli* isolates exhibited MDR phenotype, and showed high prevalence of β-lactamase and PMQR genes. Especially, one isolate harbored ESBL genes *bla*_TEM_, *bla*_SHV_, *bla*_CTX−M−9_, *bla*_CTX−M−14_, *bla*_CTX−M−64_, and carbapenemase gene *bla*_OXA−48_ and *bla*_KPC−2_, as well as PMQR genes *qnrS, qnrB, qnrD, qepA* and *aac(6')-Ib-cr*.

## Introduction

*Escherichia coli* (*E. coli*) is both a ubiquitous commensal bacterium in intestinal tract and an important pathogen of diarrhea or extraintestinal infections of humans and animals, and both commensal and pathogenic isolates usually share the same environment (Wu et al., 2013). Cephalosporins are effective for gram-negative bacterial infections, especially for infections caused by multidrug resistant (MDR) *E. coli* (Silva-Sanchez et al., 2013). At present, extended-spectrum β-lactams are not the first-line treatment in food animals, whereas the resistance to β-lactams, especially to the third- and fourth-generation cephalosporins has increased markedly accompanying their massive or inappropriate use over the past decades, and it is also considered as an important public health challenge (Agersø and Aarestrup, 2013). Nowadays, one of the most worrisome resistance mechanisms to β-lactams is the emergence of extended-spectrum β-lactamases (ESBLs), which could inactivate oxyimino-β-lactams like third-generation cephalosporins and aztreonam (Liu et al., 2015). Moreover, ESBLs are generally located on the transmissible plasmids, and could be acquired between bacteria by conjugation mechanism (Cantas et al., 2015). A recent study has further suggested that ESBL-producing *E. coli* isolate, along with their antibiotic resistance genes, can spread from food animals and animals-derived foods to humans via food-chain (Geser et al., 2012). Additionally, plasmid-mediated AmpC β-lactamase *bla*_CMY−2_, carbapenemases *bla*_NDM−1_, *bla*_OXA−48_ and *bla*_KPC−2_ are also increasingly described (Conceição-Neto et al., 2017; Subirats et al., 2017). As a result, the dissemination of ESBL-producing isolates poses a serious risk to both animal and human health. Furthermore, ESBL producers have been associated with resistance to non-β-lactam antimicrobials, such as fluoroquinolones, aminoglycosides and sulfonamides, which are often used long term to treat and prevent diseases on pig farms in China (Tian et al., 2009, 2012; Yuan et al., 2009). Especially, plasmid-mediated quinolone resistance (PMQR) genes are thought to be linked with ESBL production, and spread of *E. coli* co-expressing PMQRs and ESBLs could contribute to growing concerns about resistant *E. coli* isolates (Wang et al., 2012).

The prevalence of ESBL-producing *E. coli* isolates in food animals has been increasing worldwide, and they pose a serious challenge in controlling bacterial diarrhea in swine industry. However, very little data have been reported on the occurrence and various types of β-lactamases among *E. coli* from swine in Northwest China. The main purpose of this study was to screen ESBL-producing *E. coli* isolates collected from pig farms in Northwest China, and further analyze ESBL producers based on genetic relatedness, virulence profiles, and the occurrence and transferability of β-lactamase and PMQR genes.

## Materials and methods

### Sample collection and bacterial culture

During May 2015 to June 2017, 456 *E. coli* isolates (270 from healthy pigs, 186 from diarrheal pigs) were isolated from fecal samples of different swine in ten pig farms, which are widely dispersed across Shaanxi and Gansu provinces. Fecal samples were collected from individual pigs using a sterile cotton swab and transported to laboratory within 12 h. All samples were immediately seeded on MacConkey agar (Beijing Land Bridge Technology Co., Ltd, Beijing, China). After incubation at 37°C for 18 to 24 h, three colonies with typical *E. coli* morphology (bright pink with a dimple) were randomly selected and transferred to Eosin Methylene Blue agar (Qingdao Hope Bio Technology Co., Ltd, Qingdao, Shandong, China) for further purification. Finally, the suspect *E. coli* isolates on Eosin Methylene Blue agar (green colonies with a metallic sheen) were subjected to biochemical tests (indole, methyl red, oxidase, citrate, and triple sugar iron) as described previously (Liu et al., 2017). All confirmed *E. coli* isolates were stored at −80°C in Tryptic Soy broth medium containing 30% glycerol for later study.

### Antimicrobial susceptibility testing

The minimum inhibitory concentrations (MICs) of ampicillin, amoxicillin-clavulanic acid, ceftiofur, cefotaxime, ceftrizxone, ceftazidime, meropenem, enrofloxacin, ciprofloxacin, florfenicol, sulfamethoxazole/trimethoprim, gentamicin, amikacin, oxytetracycline, and colistin were determined by a standardized microdilution method following CLSI guidelines (CLSI, 2013). All MIC determinations were performed in triplicate, with *E. coli* ATCC 25922 serving as a quality control. Meanwhile, double-disk diffusion method was used to screen for the ESBL production among all isolates with cefotaxime and ceftazidime alone and in combination with clavulanic acid by using the guidelines recommended by CLSI (2013). Initial screening analyses indicated that 44 (9.6%) *E. coli* isolates were identified as phenotypic ESBL producers, which were further investigated for molecular characterization.

### Phylogenetic grouping and virulence genotyping

DNA from each ESBL producer was extracted using boiling method, and the distribution of phylogenetic groups of ESBL producers were determined by quadruplex PCR as described by Clermont et al. (Clermont et al., 2013). Meanwhile, enterotoxins (*elt, estA, estB, stx1, stx2*, and *astA*) and fimbrial adhesins (F4, F5, F6, F17, F18 and F41) as well as intimin encoded by *eae* gene were detected using single or multiplex PCR with specific primers as previously described (Boerlin et al., 2005; Toledo et al., 2012). The *E. coli* strains used as positive controls were B2 (*eae, stx1, stx2*), 256 (*estA, estB*), 281 (*elt*), G2077 (F4), B21523 (F5), J7203349 (F6), 320 (F41), and B37429 (F18), and *E. coli* K12 C600 was used as a negative control. Part of control strains were kindly supplied by Dr. Boothe (Auburn University, USA). The primer sequences used for PCR detection are listed in Table S1.

### Identification of β-lactamase genes and plasmid-mediated quinolone resistance genes

The occurrence of β-lactamase genes (*bla*_TEM_, *bla*_SHV_, *bla*_CTX−Ms_), plasmid-mediated AmpC β-lactamase (*bla*_CMY−2_) and carbapenemase genes (*bla*_KPC−2_, *bla*_NDM−1_, and *bla*_OXA−48_) among ESBL producers were determined by PCR and sequencing using specific primers (Table S2). The PCR products were purified using a PCR Purification Kit (TianGen, Beijing, China), and then the amplified products were sequenced by Sangon Biotech (Shanghai, China). DNA Sequences were compared with known sequences available from the BLAST program (https://blast.ncbi.nlm.nih.gov/Blast.cgi) (Altschul et al., 1997). Additionally, all ESBL producers were screened for the presence of PMQR genes (*qnrA, qnrB, qnrC, qnrD, qnrS, aac(6')-Ib-cr, oqxAB*, and *qepA*) as described previously (Liu et al., 2012; Xu et al., 2015). *E. coli* J53 strains containing pMG252, pMG298, pMG306, and pMG298 were used as positive controls for *qnrA, qnrB, qnrS*, and *aac(6*′*)-Ib-cr* genes, respectively. *E. coli* J7261205 (pSTVqepA) and S5314175 were included as positive controls for *qepA* and *oqxAB*, respectively. The positive control strain for *qnrC* was not available.

### Conjugation experiments

In order to analyze the horizontal transferability of β-lactamase and PMQR genes, especially *bla*_OXA−48_ gene, conjugation experiments were performed with eight ESBL-producing *E. coli* isolates, including four *bla*_OXA−48_ positive isolates, from different pig farms in seven different regions. Conjugation experiments were conducted by broth mating method using *E. coli* J53 AZ^r^ as a recipient (Shaheen et al., 2011). Transconjugants were selected on Tryptic Soy agar plates containing sodium azide (150 μg/ml) and cefotaxime (2 μg/ml). All transconjugants, recipient and donors were subjected to antimicrobial susceptibility testing. PCR and sequencing were performed to verify the transferability of PMQR and β-lactamase genes.

### Multilocus sequence typing (MLST)

MLST of ESBL-producing *E. coli* isolates was performed as described previously (Wirth et al., 2006). A detailed scheme of gene amplification, allelic type and sequence type assignment methods is available on the MLST website (http://mlst.warwick.ac.uk/mlst/dbs/Ecoli).

### Statistical analysis

Significance was determined by Pearson's Chi-squared test with Yates continuity correction using “R” software (version 3.0.1), and the level of significance was set at *P* < 0.05.

## Results

### Antimicrobial susceptibility of *E. coli* isolates

The results of antibiotic resistance profiles of 456 *E. coli* isolates are listed in Table 1, 96.1% of the *E. coli* isolates were resistant to ampicillin, followed by amoxicillin-clavulanic acid (91.2%), sulfamethoxazole/trimethoprim (82%), oxytetracycline (74.3%), enrofloxacin (70%), gentamicin (61.4%), florfenicol (58.8%), ciprofloxacin (57.9%), and amikacin (52.2%). The percentage of resistance to other antibacterial agents were lower than 50%. It is noteworthy that significantly more *E. coli* isolates from diarrheal pigs than those from healthy pigs were resistant to most antimicrobials tested (*P* < 0.001) with the exception of ampicillin, sulfamethoxazole/trimethoprim and colistin (Table 1). Of 456 *E. coli* isolates investigated, 44 isolates (9.6%, six isolates from healthy pigs and 38 from diarrhea pigs) were confirmed as phenotypic ESBL producers, and exhibited MDR phenotype. 97.7% of the ESBL producers were resistant to ampicillin, followed by oxytetracycline (93.2%), amoxicillin-clavulanic acid (93.2%), enrofloxacin (93.2%), sulfamethoxazole/trimethoprim (90.9%), ceftazidime (86.4%), cefotaxime (84.1%) and gentamicin (81.8%).

**Table 1:** Antibiotic resistance profiles of *E. coli* isolates from swine in Northwest China.

**Antimicrobials**	**Number of resistant isolates (%)**	***E. coli*** **isolates from healthy pigs (*****n*** = **270)**				***E. coli*** **isolates from diarrheal pigs (*****n*** = **186)**				**ESBL-producing isolates (n** = **44)**				***P*-value Isolates from healthy pigs *vs*. Isolates from diarrheal pigs/ESBL-producing isolates**
		**MIC (**μ**g/ml)**				**MIC (**μ**g/ml)**				**MIC (**μ**g/ml)**				
		**Range**	**Number of resistance (%)**	**MIC_50_**	**MIC_90_**	**Range**	**Number of resistance (%)**	**MIC_50_**	**MIC_90_**	**Range**	**Number of resistance (%)**	**MIC_50_**	**MIC_90_**	
Ampicillin	438(96.1)	1–256	252(93.3)	64	256	1–>512	186(100)	256	512	4–>512	43(97.7)	256	512	>0.05
Amoxicillin	416(91.2)	1–512	238(88.1)	32	256	1–>512	178(95.7)	128	512	1–>512	41(93.2)	256	512	< 0.01
Ceftiofur	177(38.8)	0.063–64	56(20.7)	0.5	16	1–512	121(65.1)	64	128	2–512	32(72.7)	128	256	< 0.001
Cefotaxime	184(40.4)	0.25–128	51(18.9)	0.25	32	0.5–512	133(71.5)	64	256	1–512	37(84.1)	128	256	< 0.001
Ceftazidime	188(41.2)	0.5–256	58(21.5)	2	32	2–512	130(69.9)	322	256	2–512	38(86.4)	64	256	< 0.001
Ceftrizxone	165(36.2)	0.125–128	48(17.8)	0.52	32	0.5–512	117(62.9)	64	128	1–512	30(68.2)	128	256	< 0.001
Meropenem	3(0.7)	0.03–4	0(0)	0.125	0.5	0.03–16	3(1.6)	0.25	1	0.03–16	3(6.8)	0.25	1	< 0.01
Enrofloxacin	319(70)	0.125–256	150(55.6)	16	64	0.063–512	169(90.9)	128	512	2–512	41(93.2)	128	512	< 0.001
Ciprofloxacin	264(57.9)	0.063–128	128(47.4)	8	32	0.063–512	136(73.1)	64	256	1–512	33(75)	128	256	< 0.001
Florfenicol	268(58.8)	1–256	124(45.9)	16	64	8–>512	144(77.4)	256	512	8–>512	35(79.5)	256	512	< 0.001
Gentamicin	280(61.4)	0.125–128	134(49.6)	16	128	0.5–512	146(78.5)	64	256	4–512	36(81.8)	128	256	< 0.001
Amikacin	238(52.2)	0.063–64	121(44.8)	8	64	0.063–512	117(62.9)	64	256	4–512	31(70.5)	128	256	< 0.001
Oxytetracycline	339(74.3)	1–512	169(62.6)	32	256	1–>512	170(91.4)	256	512	2–>512	41(93.2)	256	512	< 0.001
Colistin	1(0.2)	0.03–0.5	0(0)	0.03	0.125	0.063–8	1(0.5)	0.125	0.5	0.063–8	1(2.3)	0.125	0.5	>0.05
Sulfamethoxazole	374(82)	0.25–256	214(79.3)	64	256	1–512	160(86.0)	32	256	2–512	40(90.9)	128	256	>0.05

### Phylogenetic typing and virulence genotyping

Phylogenetic group analysis for 44 ESBL producers revealed that the predominant phylogenetic group was D (14/44, 31.8%), followed by phylogenetic groups B2 (11/44, 25%), A (9/44, 20.5%), B1 (6/44, 13.6%), C (3/44, 6.8%), and E (1/44, 2.3%) (Table 2). Groups D and B2 accounted for 56.8% of the ESBL producers. The frequencies of major virulence genes are listed in Table 2. 93.2% of the ESBL producers possessed at least one virulence gene. *estA* (*n* = 18) was the most prevalent toxin gene, followed by *estB* (*n* = 15), *astA* (*n* = 12), and *elt* (*n* = 10) genes. The most prevalent fimbrial adhesin was F4 (*n* = 18), followed by F18 (*n* = 10), F17 (*n* = 4), F5 (*n* = 3), F6 (*n* = 3) and F41 (*n* = 2). Furthermore, 86.4% (38/44) of the ESBL producers carried both enterotoxins and fimbrial adhesins, and 27 different virotypes were identified according to the combinations of enterotoxin and adhesin genes. The *eae* gene was detected in two ESBL producers (4.5%), while *stx1* and *stx2* were not detected.

**Table 2:** Extended-spectrum β-lactamase-producing *E. coli* isolates from swinein Northwest China.

**Isolate ID**	**Phylogentic group**	**Sources**	**MLST**	**Resistance profiles**	**β-lactamase genes**	**PMQR genes**	**Virulence genes**
FF170322	A	Diarrheal pig	ST10	AMP AMC EFT CAZ CEX OTC ENR CIP FFC SXT	TEM-1, CTX-M-9, CTX-M-123	*qnrA, qnrB, qepA, oqxAB*	*estA, astA*,F18
JY160633	A	Healthy pig	ST10	AMP AMC CAZ ENR CIP FFC OTC GEN	TEM-1, CTX-M-9, CTX-M-123	*qnrS, aac(6')-Ib-cr*	*estA*, F17
FP170743	A	Diarrheal pig	ST10	AMP AMC EFT CTX CEX ENR CIP FFC OTC SXT	TEM-1, CTX-M-14, CTX-M-123	*qnrS, qnrA*	*estB*, F18
FP170756	A	Diarrheal pig	ST10	AMP AMC EFT CTX CAZ CEX ENR CIP FFC OTC GEN AMK SXT	TEM-1, CTX-M-15	*qnrS, qnrA*	*astA*, F5
MX161024	A	Diarrheal pig	ST10	AMP AMC EFT CAZ ENR CIP FFC OTC GEN AMK SXT	TEM-1, CTX-M-15	*qnrA*	*astA*, F4
HX160912	A	Diarrheal pig	ST167	AMP AMC CTX ENR SXT	TEM-1, CTX-M-64	*qnrS, qnrA*	*estA*, F17
JY160509	A	Healthy pig	ST175	AMP CTX CEX OTC CAZ GEN SXT	TEM-1,CTX-M-14	*qnrA*	*estA*, F4
FP160935	A	Healthy pig	ST2715	AMP AMC CAZ ENR CIP OTC SXT	SHV-12	-	-
JY170618	A	Diarrheal pig	ST5236	AMP AMC CTX CAZ FFC GEN AMK OTC	TEM-1, CTX-M-14	*qnrA*	*estA*, F18
HX160826	B1	Diarrheal pig	ST75	AMP CAZ CEX ENR FFC OTC	TEM-1, CTX-M-14	*qnrS*	*estA*, F17
ZZ160931	B1	Healthy pig	ST155	AMP AMC CTX CAZ OTC GEN AMK SXT	TEM-1, CTX-M-64	-	-
ZZ170521	B1	Diarrheal pig	ST183	AMP AMC CTX CAZ ENR OTC SXT	TEM-1, CTX-M-14	*qnrA*	*estA, astA*, F6
FP170723	B1	Healthy pig	ST302	AMC CAZ OTC ENR FFC	CTX-M-123	-	-
JY170327	B1	Diarrheal pig	ST355	AMP CAZ OTC ENR SXT	TEM-1, CTX-M-14	*qnrS*	*eae*
FF170327	B1	Healthy pig	ST443	AMP AMC CAZ CEX ENR OTC SXT	CTX-M-14	*qnrS, aac(6')-Ib-cr*	*astA*
HX161021	B2	Diarrheal pig	ST29	AMP AMC EFT CTX CAZ ENR CIP FFC OTC GEN AMK SXT	CTX-M-14, CTX-M-123	*qnrB, aac(6')-Ib-cr*	*estA*, F4
FF170425	B2	Diarrheal pig	ST95	AMP AMC EFT CTX CAZ CEX ENR CIP FFC OTC GEN AMK SXT	TEM-1, CTX-M-15, CTX-M-64	-	*astA*, F18
MX160918	B2	Diarrheal pig	ST104	AMP AMC EFT CTX CEX OTC ENR CIP FFC GEN AMK SXT	TEM-1, CTX-M-64	*qnrS, aac(6')-Ib-cr*	*astA, estA*, F4
JY160522	B2	Diarrheal pig	ST104	AMP AMC EFT CTX CAZ OTC ENR CIP FFC GEN AMK SXT	TEM-1, CTX-M-64	*qnrS, aac(6')-Ib-cr*	*estA*, F4, F18
HX160809	B2	Diarrheal pig	ST127	AMP AMC EFT CTX CAZ CEX ENR CIP FFC OTC GEN AMK SXT	TEM-1, CTX-M-123	*qnrS*	*estB*, F6
FP170708	B2	Diarrheal pig	ST127	AMP AMC EFT CTX CEX ENR CIP FFC OTC GEN AMK SXT	CTX-M-123	-	*estB*, F5
JY160503	B2	Diarrheal pig	ST131	AMP AMC EFT CTX CAZ CEX MEM ENR CIP FFC OTC GEN AMK SXT	CTX-M-9, CTX-M-14, CTX-M-64, KPC-2, OXA-48	*qnrS, qnrB, qnrD, aac(6')-Ib-cr, qepA*	*elt, estA*, F6
MX150822	B2	Diarrheal pig	ST278	AMP AMC EFT CTX CEX ENR CIP FFC OTC GEN AMK SXT	TEM-1, CTX-M-14	*qnrS*	*astA, estB*, F4
FF170416	B2	Diarrheal pig	ST355	AMP AMC EFT CTX CAZ CEX ENR CIP FFC OTC GEN AMK SXT	SHV-2, CTX-M-64	*qnrB*	*estA*, F4
FF170325	B2	Diarrheal pig	ST372	AMP AMC EFT CTX CAZ CEX ENR CIP FFC OTC GEN AMK SXT	TEM-1, CTX-M-14, CTX-M-15, CTX-M-64	*qnrS, aac(6')-Ib-cr*	*estB*, F4, F18
FF170316	B2	Diarrheal pig	ST372	AMP AMC EFT CTX CAZ ENR CIP FFC OTC GEN AMK SXT	CTX-M-14, CTX-M-15, CTX-M-123	*qnrS, aac(6')-Ib-cr*	*elt, estA*, F4
MX150923	C	Diarrheal pig	ST23	AMP AMC CTX CAZ EFT CEX ENR FFC GEN AMK SXT	TEM-1, CTX-M-14	*qnrS, qnrA*	*estA, estB*, F18
MX150814	C	Diarrheal pig	ST23	AMP AMC EFT CTX CAZ ENR CIP OTC GEN AMK SXT	TEM-1, CTX-M-14	*qnrB, aac(6')-Ib-cr*	*elt*, F4
ZZ160815	C	Diarrheal pig	ST23	AMP AMC EFT CTX CAZ CEX ENR CIP FFC OTC GEN SXT	TEM-1, CTX-M-14	*qnrS*	*estA*, F18
JY160518	D	Diarrheal pig	ST38	AMP AMC EFT CTX CAZ CEX MEM ENR CIP FFC OTC GEN AMK SXT	TEM-1, CTX-M-15, OXA-48	*qnrS, qnrA*	*elt, estB*, F4
HX160976	D	Diarrheal pig	ST38	AMP AMC EFT CTX CAZ CEX ENR CIP FFC OTC GEN AMK SXT	CTX-M-9, CTX-M-14	*qnrA*	*elt, estB*, F41
HX160944	D	Diarrheal pig	ST38	AMP AMC EFT CTX CAZ CEX ENR CIP FFC OTC GEN AMK SXT	CTX-M-9,CTX-M-14, CTX-M-64	*qnrB*	*elt, astA*, F4
HX161006	D	Diarrheal pig	ST69	AMP AMC EFT CTX CAZ CEX ENR CIP FFC OTC GEN AMK SXT	TEM-1, CTX-M-14	*qnrS, qnrA*	*astA*, F5
MX150820	D	Diarrheal pig	ST405	AMP AMC EFT CTX CAZ CEX MEM ENR CIP FFC OTC GEN AMK SXT	SHV-12, CTX-M-14, CTX-M-15, NDM-1	*qnrB, aac(6')-Ib-cr*	*elt, estA*, F4
LZ161015	D	Diarrheal pig	ST405	AMP AMC EFT CTX CAZ ENR CIP FFC OTC GEN AMK SXT	SHV-12, CTX-M-15	*qnrS, qnrB*	*estB*, F4
JC160611	D	Diarrheal pig	ST405	AMP AMC EFT CTX CAZ CEX ENR CIP FFC OTC GEN AMK SXT	TEM-1, CTX-M-9, KPC-2	*qnrS, qnrB*	*estB*, F4
JY160512	D	Diarrheal pig	ST405	AMP AMC EFT CTX CAZ CEX ENR CIP FFC OTC GEN AMK SXT CLT	TEM-1, CTX-M-9, CTX-M-14, CTX-M-15	*qnrA, qnrD, oqxAB*	*elt, estA*, F4
FP170711	D	Diarrheal pig	ST405	AMP AMC EFT CTX CAZ CEX ENR CIP FFC OTC GEN AMK SXT	TEM-1, SHV-12, CTX-M-15, OXA-48	*aac(6')-Ib-cr, oqxAB*	*elt, estB*, F4
HX170832	D	Diarrheal pig	ST405	AMP AMC EFT CTX CAZ CEX ENR CIP FFC OTC GEN AMK SXT	TEM-1, SHV-12, CTX-M-15, OXA-48	*aac(6')-Ib-cr*	*elt, estB*, F4
SY160832	D	Diarrheal pig	ST648	AMP AMC EFT CTX CAZ CEX ENR CIP FFC OTC GEN AMK SXT	SHV-2, CTX-M-9, CTX-M-14, CTX-M-123	*qnrA, aac(6')-Ib-cr*	*estB, astA*, F17
ZZ160908	D	Diarrheal pig	ST648	AMP AMC EFT CTX CEX ENR CIP FFC OTC GEN AMK SXT	TEM-1, CTX-M-9, CTX-M-14, CTX-M-15	*qnrA*	*estB, astA*, F41
ZZ160917	D	Diarrheal pig	ST648	AMP AMC EFT CTX CAZ CEX ENR CIP FFC OTC GEN AMK SXT	TEM-1, CTX-M-14, CTX-M-15, CTX-M-64	*qnrS*	*estA*, F18
JY160865	D	Diarrheal pig	ST648	AMP AMC EFT CTX CAZ CEX ENR CIP FFC OTC GEN AMK SXT	TEM-1, CTX-M-14, CTX-M-15, CTX-M-64	*qnrS*	*estB*, F18
FP170733	E	Diarrheal pig	ST350	AMP AMC CTX CEX ENR OTC SXT	CTX-M-9	*qnrS*	*eae*

*AMP, ampicillin; AMC, amoxicillin-clavulanic acid; EFT, ceftiofur; CTX, cefotaxime; CAZ, ceftazidime; CEX, ceftriaxone; MEM, meropenem; ENR, enrofloxacin; CIP, ciprofloxacin; FFC, florfenicol, OTC, oxytetracycline; GEN, gentamicin; AMK, amikacin; SXT, sulfamethoxazole-trimethoprim; CLT, colistin*.

### Characterization of ESBL and PMQR genes

Each ESBL producer harbored at least one β-lactamase gene. *bla*_CTX−M_, *bla*_TEM_, *bla*_SHV_, *bla*_OXA−48_, *bla*_KPC−2_, and *bla*_NDM−1_ were detected in forty (90.9%), thirty (68.2%), seven (15.9%), four (9.1%), two (4.5%), and one (2.3%) isolates, respectively (Table 2). AmpC β-lactamase gene *bla*_CMY−2_ was not detected. Overall, *bla*_CTX−M−14_ (*n* = 24) was the predominant genotype in *bla*_CTX−M_ positive isolates, followed by *bla*_CTX−M−15_ (*n* = 14), *bla*_CTX−M−64_ (*n* = 11), *bla*_CTX−M−9_ (*n* = 10) and *bla*_CTX−M−123_ (*n* = 9), while *bla*_CTX−M−1_ gene was not detected. The distribution of PMQR genes among 44 ESBL-producing *E. coli* isolates is shown in Table 2. 88.6% (39/44) of ESBL producers were found to harbor at least one PMQR gene, and seven types of PMQR were identified. *qnrS, qnrA, aac(6')-Ib-cr, qnrB, oqxAB, qnrD*, and *qepA* were detected alone or incombination in 52.3% (24/44), 34.1% (15/44), 27.3% (12/44), 20.5% (9/44), 6.8% (3/44), 4.5% (2/44), and 4.5% (2/44) of ESBL-producing isolates, respectively. *qnrS* was the most common PMQR gene, and *qnrS*+*qnrA* was the most common combination (*n* = 6). No isolates were positive for *qnrC* gene. Among 39 PMQR positive isolates, 28 (80%) isolates were positive for more than one PMQR determinant. Furthermore, all PMQR genes were detected in co-existence with β-lactamases, and one isolate from the intestinal content of a 15-day-old dead piglet with serious diarrhea harbored β-lactamase genes *bla*_TEM_, *bla*_SHV_, *bla*_CTX−M−9_, *bla*_CTX−M−14_, *bla*_CTX−M−64_ and carbapenemase gene *bla*_OXA−48_ and *bla*_KPC−2_, as well as PMQR genes *qnrS, qnrB, qnrD, qepA*, and *aac(6')-Ib-cr*. *bla*_OXA−48_ gene was detected in four meropenem-non-susceptible or meropenem-resistant isolates.

### Conjugation experiments

Five out of eight ESBL producers successfully transferred the β-lactamase genes to recipient strain *E. coli* J53 AZ^r^. PCR analysis showed that the presence of respective β-lactamase genes, including one *bla*_OXA−48_-carrying plasmids from all transconjugants. Accordingly, PMQR genes *qnr* and *aac-(6*′*)-Ib-cr* were co-transferred with β-lactamase genes (Table 3). Antimicrobial susceptibility patterns showed that all donors and their transconjugants were resistant to amoxicillin-clavulanic acid, ampicillin, ceftiofur, cefotaxime, and all transconjugants exhibited an increase of at least 8-fold in MICs compared to the recipient, *E. coli* J53 AZ^r^. The enrofloxacin MICs for four transconjugants harboring *aac-(6*′*)-Ib-cr* ranged from 0.125 to 0.5 mg/L, representing an increase of 4-fold to 8-fold compared with the recipient (Table 3). Additionally, the transconjugants remained susceptible to meropenem, enrofloxacin, florfenicol, oxytetracycline, gentamicin, sulfamethoxazole-trimethoprim and colistin, whereas one *bla*_OXA−48_ positive transconjugant reduced meropenem susceptibility.

**Table 3:** Antimicrobial susceptibility profiles of extended-spectrum β-lactamase-producing *E. coli* isolates used in the conjugation experiments.

**Isolates**	**MIC (**μ**g/ml) of antimicrobials**															**Presence or absence of**					
	**AMP**	**AMC**	**EFT**	**CTX**	**CAZ**	**CEX**	**MEM**	**ENR**	**FFC**	**OTC**	**GEN**	**SXT**	**CLT**	**TEM**	**SHV**	**CTX-M-15**	**CTX-M-14**	**CTX-M-9**	**OXA-48**	***qnr***	***aac(6′)-Ib-cr***
**DONORS**																					
JY160503	512	32	64	32	64	32	8	128	64	128	32	128	0.063				+	+	+	+	+
JY160512	256	32	64	32	128	8	0.03	64	128	64	32	256	4	+		+	+	+		+	
FP170711	256	64	64	64	256	32	16	128	128	64	64	256	0.125	+	+	+			+		+
ZZ160908	256	64	128	128	1	64	0.063	128	64	128	32	128	0.125	+		+	+	+		+	
MX150820	256	32	64	16	2	32	4	256	64	64	64	128	0.25		+	+	+			+	+
Recipient J53AZ^r^	4	1	0.125	0.125	0.063	0.063	0.015	0.063	0.063	0.25	0.25	0.063	0.03								
**TRANSFORMANTS**																					
Trans-JY160503	256	64	64	16	64	32	0.5	0.5	0.063	0.125	0.125	0.125	0.03				+	+		+	+
Trans-JY160512	256	32	32	32	128	4	0.03	0.25	0.125	0.5	0.25	0.125	0.063	+		+	+	+		+	
Trans-FP170711	128	32	64	16	128	2	0.5	0.125	0.063	0.5	0.25	0.25	0.03	+	+				+		+
Trans-ZZ160908	256	32	128	64	0.5	16	0.03	0.25	0.03	0.25	0.5	0.063	0.03	+		+	+	+		+	
Trans-MX150820	128	32	64	16	0.5	32	0.03	0.5	0.03	0.125	0.125	0.063	0.063		+	+	+				+

### MLST profiles

Forty-four ESBL producers belonged to 24 sequence types (STs) (Table 2). The most prevalent was ST405 (*n* = 6), followed by ST10 (*n* = 5). ST405 (*n* = 6), ST648 (*n* = 4) and ST38 (*n* = 3) of phylogenetic group D accounted for 29.5% of the ESBL producers. The carbapenemases *bla*_OXA−48_, *bla*_NDM−1_ and *bla*_KPC−2_ were connected with sequence types ST405, ST131 and ST38. The isolates with same STs have similar virotypes and β-lactamase profiles.

## Discussion

The prevalence of ESBL-producing *E. coli* isolates in food animals has been increasing worldwide (Liebana et al., 2013). In China, diarrhea caused by pathogenic *E. coli*, especially ESBL-producing *E. coli* poses a serious threat to the swine industry and public health (Lei et al., 2010; Xu et al., 2015). The present study is the first contribution to explore the detailed characterizations of ESBL-producing *E. coli* isolates from pigs in Northwest China. Forty-four (9.6%) isolates were confirmed as ESBL producers, while it is noteworthy that 456 *E. coli* in this study isolated from feces of healthy and diarrheal pigs, and the prevalence of ESBL producer were significantly higher among isolates from diarrheal pigs than that form healthy isolates (20.4vs. 2.2%; *P* < 0.001). The detectable rate of ESBL producer in diarrheal pigs was similar with the result in Sichuan (26.8%), a neighbor province of Shaanxi, while it was significantly lower than in Heilongjiang (43.2%), a province in the northeast China (Tian et al., 2009; Xu et al., 2015). Moreover, our results showed that ESBL producers mainly belonged to phylogenetic groups D and B2, and to a lesser extent to phylogenetic A, while the previous studies showed that *E. coli* from pigs or duck in China also mainly fell into phylogenetic groups A (Wang et al., 2010; Ma et al., 2012). It is further confirmed that the emergence of ESBL-producing *E. coli* has a geographic variation with respect to demographic, environmental, behavioral, socioeconomic and infectious risk factors with the extending of ESBL-producing isolates stage by stage.

All 456 *E. coli* isolates in this study were tested for their susceptibility to 15 antimicrobial agents. Overall, the number of resistant isolates in ESBL producers and isolates from diarrheal pigs were higher than that from healthy pigs (*P* > 0.001). It is suggested that the isolates from diarrheal pigs may be more likely to develop antibiotic resistance than that from healthy pigs because of the frequent use of antimicrobials in preventing and treating diarrhea. All ESBL producers were resistant to at least five antimicrobial agents, and vast majority of them (>93%) remained susceptible to meropenem and colistin, which are considered the effective candidates for treatment of serious infections caused by *E. coli* in pig farms of China. According to the virotypes, 86.4% of ESBL producers carried both enterotoxins and fimbrial adhesins. It is indicated that these isolates should be enterotoxigenic *E. coli* (ETEC), which are responsible for neonatal diarrhea and postweaning diarrhea in piglets. F4 fimbrial adhesin was present 40.9% of the ESBL producers, it is consistent with the previous studies that F4 adhesin gene is one of the most frequently found genes in *E. coli* isolates from suckling and weaning piglets (Vu Khac et al., 2006; Zhang et al., 2007). Furthermore, the gene combinations of F4+*estA*/*estB* were present in 34.1% of the isolates.

Since the early 2000s, CTX-M-type ESBLs have been increasingly reported, and they have now replaced TEM and SHV as the most common type of ESBL (Barguigua et al., 2011). The most predominant ESBL gene in this study was *bla*_CTX−M_ (90.9%), and the similar findings showed that CTX-Ms accounted for 87.1% of ESBL-producing *E. coli* isolated from food animals based on a previous survey in China (Rao et al., 2014). *bla*_CTX−M−14_ remained the most common genotype, and followed by *bla*_CTX−M−15_. It is surprising that no isolate contained *bla*_CTX−M−1_, whereas it was detected in the ESBL-producing *E. coli* from dogs, retail pork and water bodies in Shaanxi province (Xi et al., 2015; Liu et al., 2016b). In regards to the linkage of phylogenetic group and β-lactamases, isolates of group D harbored more β-lactamases genes, and isolates of group A harbored less β-lactamases. Novel hybrid β-lactamase gene *bla*_CTX−M−123_ was firstly discovered in *E. coli* from pig feces in China in 2013 (He et al., 2013), and it was detected in nine ESBL producers in this study. Moreover, four ESBL producers were commensal isolates from healthy pigs, it was further indicated that some commensal organisms in animals have acquired β-lactamase genes with the increasing use of β-lactams in animals. *bla*_OXA−48_ was detected in four ESBL producers from diarrheal pigs. As a globally emerging carbapenemase gene, *bla*_OXA−48_ could hydrolyze carbapenems and β-lactamase inhibitors but has no activity toward broad-spectrum cephalosporins (Mathers et al., 2013). *bla*_OXA−48_ was firstly discovered in *E. coli* from dogs in Germany in 2013, and afterward it was reported in *E. coli* from companion animals in the United States in 2016 (Stolle et al., 2013; Liu et al., 2016a). In 2017, it was reported in pigs in Italy (Pulss et al., 2017). Most recently, it was detected in *Enterobacteriaceae* from river water in Algeria (Tafoukt et al., 2017). Considering the importance of *bla*_OXA−48_ gene in public health, it is necessary to further investigate the dissemination of *bla*_OXA−48_ producing *E. coli* isolates among different sources.

PMQR genes were often found to be strongly associated with β-lactamase genes and even in the same plasmid, and they are not merely able to confer resistance against quinolones but also often related to ESBLs (Jeong et al., 2011). In this study, PMQR genes were present in 88.6% of ESBL producers, and the similar findings have been reported in ESBL-producing *E. coli* isolates form pigs in previous studies in China by Xu et al. (87.4%) and Liu et al (83.8%) (Liu et al., 2013; Xu et al., 2015). Thirty-seven ESBL producers (84.1%) harbored at least one *qnr* gene, and *qnrS* was the predominant, whereas a low prevalence of *qnr* genes was detected among ESBL-producing *E. coli* isolates in France and Canada (1.6 and 1%, respectively). In addition, *qepA* gene was detected in combination with other PMQR and β-lactamase genes in four isolates (10%). The frequent combination of β-lactamases and PMQRs in this study further supported the previous studies that coproduction of β-lactamase and PMQR genes could contribute to the dissemination of MDR isolates, and also reflect the fact that genes encoding resistance to β-lactams and quinolones are located on the same plasmid.

Twenty-four different sequence types were identified, and three sequence types (ST405, ST10, and ST648) accounted for 34.1% of the ESBL producers. Sequence types ST10, ST38, ST131, ST648, and ST405 clones were documented in different sources according to MLST databases, and could favor the dissemination of CTX-M worldwide among *E. coli* isolates (Hernandez and Gonzalez-Acuna, 2016). In the present study, a few isolates belonging to different STs shared similar β-lactamase and PMQR gene profiles, whereas several isolates belonging to same ST exhibited different gene profiles. The similar results were observed among *E. coli* isolates from dogs and cats in previous studies (Liu et al., 2016a,b). The possible explanation is that the pig trade, personnel exchanges and water sources among adjacent regions may lead to the dissemination of isolates with same gene profiles or same ST types. Anyway, deeper analyses for such isolates are necessary in the future. It is noteworthy that *bla*_OXA−48_ gene were detected in four isolates with reduced susceptibility or resistance to meropenem. The *bla*_OXA−48_ positive isolates co-harbored variants of β-lactamase genes, and they also were associated with sequence types ST38, ST405, and ST131. Additionally, *bla*_OXA−48_ positive *E. coli* clone ST38 had been previously reported in France, Germany and Algeria (Poirel et al., 2011; Kaase et al., 2016; Bouaziz et al., 2017). In the current study, we firstly reported the occurrence of *bla*_OXA−48_ positive *E. coli* clone ST38 from a sucking piglet with diarrhea in Shaanxi. Clone ST38 has achieved notoriety as it is now rapidly and globally disseminated, and its potential to serve as a vehicle for spread of carbapenemases is profoundly alarming. *bla*_NDM−1_ producing *E. coli* isolate, emerging as a public health threat, has gained global attention as it could hydrolyze almost all β-lactams with the exception of aztreonam (Nordmann et al., 2012), and it has previously been detected in *E. coli* isolates from pigs (Fischer et al., 2012). Our results revealed that *bla*_NDM−1_ and other β-lactamase genes coexisted in one isolates, it is a potential public health concern as the pig carrying *bla*_NDM−1_ and other β-lactamase genes may enter the food chain.

## Conclusion

In conclusion, all ESBL-producing *E. coli* isolates both from healthy and diarrheal pigs in Northwest China exhibited MDR phenotype. The *bla*_CTX−M−14_ and *qnrS* were the predominant β-lactamase gene and PMQR gene in ESBL producers, respectively. *estA* and F4 were the most prevalent enterotoxin and fimbrial adhesin, respectively. One ST131 isolate harbored β-lactamase genes *bla*_TEM_, *bla*_SHV_, *bla*_CTX−M−9_, *bla*_CTX−M−14_, *bla*_CTX−M−64_, and carbapenemase genes *bla*_OXA−48_ and *bla*_KPC−2_, as well as PMQR genes *qnrS, qnrB, qnrD, qepA* and *aac(6')-Ib-cr*. The findings could provide useful information for a national monitoring of antimicrobial resistance in bacteria from food animals in China.

## Author contributions

XL conceived and designed the experiments. HL and LW designed the experiment and drafted the manuscript. XL, HL, LW, HZ, and QP performed the experiments. XL, YL, and QL analyzed and explained the data for the work. All authors critically revised and approved the final manuscript.

### Conflict of interest statement

The authors declare that the research was conducted in the absence of any commercial or financial relationships that could be construed as a potential conflict of interest.
