# Supplementary material for: Molecular Characterization of Extended-Spectrum β-Lactamase-Producing Multidrug Resistant Escherichia coli From Swine in Northwest China
Source: Front Microbiol. 2018 Aug 3;9:1756. doi: 10.3389/fmicb.2018.01756 (PMC6085443; doi:10.3389/fmicb.2018.01756)
Supplement: Supplementary file 1 [file Table_1.DOCX]

**Table S1** The Oligonucleotide primers used in this study

| **Target gene** | **Primer sequence (5’-3’)** | **Fragment size (bp)** | **Reference** |
| --- | --- | --- | --- |
| *elt* | ATTTACGGCGTTACTATCCTC/ TTTTGGTCTCGGTCAGATATG | 280 | ([Osek et al., 1999](#_ENREF_9)) |
| *astA* | CCATCAACACAGTATATCCGA/ GGTCGCGAGTGACGGCTTTGT | 111 | ([Yamamoto and Nakazawa, 1997](#_ENREF_14)) |
| *stx1* | ATAAATCGCCATTCGTTGACTAC/ AGAACGCCCACTGAGATCATC | 180 | ([Fagan et al., 1999](#_ENREF_4)) |
| *stx2* | GGCACTGTCTGAAACTGCTCC/ TCGCCAGTTATCTGACATTCTG | 255 | ([Fagan et al., 1999](#_ENREF_4)) |
| *eae* | ATCTTCTGCGTACTGCGTTCA/ CATTATGGAACGGCAGAGGT | 790 | ([Beaudry et al., 1996](#_ENREF_1)) |
| *estA* | TCCCCTCTTTTAGTCAGTCAACTG/ GCACAGGCAGGATTACAACAAAGT | 171 | ([Ngeleka et al., 2003](#_ENREF_7)) |
| *estB* | GCAATAAGGTTGAGGTGAT/ GCCTGCAGTGAGAAATGGAC | 377 | ([Lortie et al., 1991](#_ENREF_6)) |
| F4 | GGTCGCGAGTGACGGCTTTGT/ CCACTGAGTGCTGGTAGTTACAGCC | 792 | ([Vu Khac et al., 2006](#_ENREF_13)) |
| F5 | TGCGACTACCAATGCTTCTG/ TATCCACCATTAGACGGAGC | 450 | ([Ojeniyi et al., 1994](#_ENREF_8)) |
| F6 | TCTGCTCTTAAAGCTACTGG/ AACTCCACCGTTTGTATCAG | 333 | ([Ojeniyi et al., 1994](#_ENREF_8)) |
| F18 | GTGAAAAGACTAGTGTTTATTTC/ CTTGTAAGTAACCGCGTAAGC | 510 | ([Imberechts et al., 1997](#_ENREF_5)) |
| F17 | GGGCTGACAGAGGAGGTGGGGC/ CCCGGCGACAAC TTC ATC ACC GG | 411 | ([Vu Khac et al., 2006](#_ENREF_13)) |
| F41 | GAGGGACTTTCATCTTTTAG/ AGTCCATTCCATTTATAGGC | 431 | ([Ojeniyi et al., 1994](#_ENREF_8)) |
| CTX-M-1 group | CCCATGGTTAAAAAATCACTGC/ CAGCGCTTTTGCCGTCTAAG | 942 | ([Yu et al., 2007](#_ENREF_16)) |
| CTX-M-2 group | CGACGCTACCCCTGCTATT/ CCAGCGTCAGATTTTTCAGG | 552 | ([Sun et al., 2010](#_ENREF_12)) |
| CTX-M-8 group | AACACGCAGACGCTCTAC/ TCGAGCCGGAAGGTGTCAT | 326 | ([Dallenne et al., 2010](#_ENREF_3)) |
| CTX-M-9 group | ATGGTGACAAAGAGAGTGCAAC/ TTACAGCCCTTCGGCGATGATT | 876 | ([Zhang et al., 2014](#_ENREF_17)) |
| TEM | TTCTTGAAGACGAAAGGGC/ ACGCTCAGTGGAACGAAAAC | 1,150 | ([Brinas et al., 2002](#_ENREF_2)) |
| SHV | CACTCAAGGATGTATTGTG/ TTAGCGTTGCCAGTGCTCG | 850 | ([Brinas et al., 2002](#_ENREF_2)) |
| CMY-2 | ATGATGAAAAAATCGTTATGC/ TTGCAGCTTTTCAAGAATGCGC | 1143 | ([Yan et al., 2004](#_ENREF_15)) |
| KPC-2 | CGTCTAGTTCTGCTGTCTTG/ CTTGTCATCCTTGTTAGGCG | 798 | ([Poirel et al., 2011a](#_ENREF_10)) |
| NDM-1 | GGTTTGGCGATCTGGTTTTC/ CGGAATGGCTCATCACGATC | 699 | ([Poirel et al., 2011a](#_ENREF_10)) |
| OXA-48 | GCGTGGTTAAGGATGAACAC/ CATCAAGTTCAACCCAACCG | 438 | ([Poirel et al., 2011b](#_ENREF_11)) |

**References**

Beaudry, M., Zhu, C., Fairbrother, J.M., and Harel, J. (1996). Genotypic and phenotypic characterization of *Escherichia coli* isolates from dogs manifesting attaching and effacing lesions. *Journal of Clinical Microbiology* 34**,** 144-148.

Brinas, L., Zarazaga, M., Saenz, Y., Ruiz-Larrea, F., and Torres, C. (2002). Beta-lactamases in ampicillin-resistant *Escherichia coli* isolates from foods, humans, and healthy animals. *Antimicrob Agents Chemother* 46**,** 3156-3163.

Dallenne, C., Da Costa, A., Decre, D., Favier, C., and Arlet, G. (2010). Development of a set of multiplex PCR assays for the detection of genes encoding important beta-lactamases in *Enterobacteriaceae*. *J Antimicrob Chemother* 65**,** 490-495.

Fagan, P.K., Hornitzky, M.A., Bettelheim, K.A., and Djordjevic, S.P. (1999). Detection of shiga-like toxin (*stx1* and *stx2*), intimin (*eaeA*), and enterohemorrhagic *Escherichia coli* (EHEC) hemolysin (EHEC *hlyA*) genes in animal feces by multiplex PCR. *Appl Environ Microbiol* 65**,** 868-872.

Imberechts, H., Bertschinger, H.U., Nagy, B., Deprez, P., and Pohl, P. (1997). Fimbrial colonisation factors F18ab and F18ac of *Escherichia coli* isolated from pigs with postweaning diarrhea and edema disease. *Adv Exp Med Biol* 412**,** 175-183.

Lortie, L.A., Dubreuil, J.D., and Harel, J. (1991). Characterization of *Escherichia coli* strains producing heat-stable enterotoxin b (STb) isolated from humans with diarrhea. *J Clin Microbiol* 29**,** 656-659.

Ngeleka, M., Pritchard, J., Appleyard, G., Middleton, D.M., and Fairbrother, J.M. (2003). Isolation and association of *Escherichia coli* AIDA-I/STb, rather than EAST1 pathotype, with diarrhea in piglets and antibiotic sensitivity of isolates. *Journal of Veterinary Diagnostic Investigation* 15**,** 242-252.

Ojeniyi, B., Ahrens, P., and Meyling, A. (1994). Detection of fimbrial and toxin genes in *Escherichia coli* and their prevalence in piglets with diarrhoea. The application of colony hybridization assay, polymerase chain reaction and phenotypic assays. *Zentralbl Veterinarmed B* 41**,** 49-59.

Osek, J., Gallien, P., Truszczynski, M., and Protz, D. (1999). The use of polymerase chain reaction for determination of virulence factors of *Escherichia coli* strains isolated from pigs in Poland. *Comp Immunol Microbiol Infect Dis* 22**,** 163-174.

Poirel, L., Walsh, T.R., Cuvillier, V., and Nordmann, P. (2011a). Multiplex PCR for detection of acquired carbapenemase genes. *Diagn Microbiol Infect Dis* 70**,** 119-123.

Poirel, L., Walsh, T.R., Cuvillier, V., and Nordmann, P. (2011b). Multiplex PCR for detection of acquired carbapenemase genes. *Diagnostic Microbiology and Infectious Disease* 70**,** 119-123.

Sun, Y., Zeng, Z., Chen, S., Ma, J., He, L., Liu, Y., Deng, Y., Lei, T., Zhao, J., and Liu, J.H. (2010). High prevalence of bla(CTX-M) extended-spectrum beta-lactamase genes in *Escherichia coli* isolates from pets and emergence of CTX-M-64 in China. *Clinical Microbiology and Infection* 16**,** 1475-1481.

Vu Khac, H., Holoda, E., Pilipcinec, E., Blanco, M., Blanco, J.E., Mora, A., Dahbi, G., Lopez, C., Gonzalez, E.A., and Blanco, J. (2006). Serotypes, virulence genes, and PFGE profiles of *Escherichia coli* isolated from pigs with postweaning diarrhoea in Slovakia. *BMC Vet Res* 2**,** 10.

Yamamoto, T., and Nakazawa, M. (1997). Detection and sequences of the enteroaggregative Escherichia coli heat-stable enterotoxin 1 gene in enterotoxigenic *E. coli* strains isolated from piglets and calves with diarrhea. *J Clin Microbiol* 35**,** 223-227.

Yan, J.J., Hong, C.Y., Ko, W.C., Chen, Y.J., Tsai, S.H., Chuang, C.L., and Wu, J.J. (2004). Dissemination of blaCMY-2 among *Escherichia coli* isolates from food animals, retail ground meats, and humans in southern Taiwan. *Antimicrob Agents Chemother* 48**,** 1353-1356.

Yu, Y., Ji, S., Chen, Y., Zhou, W., Wei, Z., Li, L., and Ma, Y. (2007). Resistance of strains producing extended-spectrum beta-lactamases and genotype distribution in China. *J Infect* 54**,** 53-57.

Zhang, J., Zheng, B., Zhao, L., Wei, Z., Ji, J., Li, L., and Xiao, Y. (2014). Nationwide high prevalence of CTX-M and an increase of CTX-M-55 in *Escherichia coli* isolated from patients with community-onset infections in Chinese county hospitals. *BMC Infect Dis* 14**,** 659.
