# Supplementary material for: Molecular Characterization of Extended-Spectrum β-Lactamase-Producing Multidrug Resistant Escherichia coli From Swine in Northwest China
Source: Front Microbiol. 2018 Aug 3;9:1756. doi: 10.3389/fmicb.2018.01756 (PMC6085443; doi:10.3389/fmicb.2018.01756)
Supplement: Supplementary file 2 [file Table_2.docx]

Table S2 Extended-spectrum β-lactamase-producing *E. coli* isolates from swine in Northwest China

| **Isolate ID** | **Location** | **Phylogentic group** | **Sources** | **MLST** | **MIC (μg/ml)** | | | | | | | | | | | | | | | **β-lactamase genes** | **PMQR genes** | **Virulence genes** |
| --- | --- | --- | --- | --- | --- | --- | --- | --- | --- | --- | --- | --- | --- | --- | --- | --- | --- | --- | --- | --- | --- | --- |
|  |  |  |  |  | **AMP** | **AMC** | **EFT** | **CTX** | **CAZ** | **CEX** | **MEM** | **ENR** | **CIP** | **FFC** | **OTC** | **GEN** | **AMK** | **SXT** | **CLT** |  |  |  |
| FF170322 | Fufeng | A | Diarrheal pig | ST10 | >512 | 128 | 8 | 16 | 32 | 4 | <.0.03 | 16 | 32 | 32 | 128 | 2 | 1 | 128 | <.0.03 | TEM-1, CTX-M-9, CTX-M-123 | *qnrA*, *qnrB*, *qepA, oqxAB* | *estA*, *astA*,F18 |
| JY160633 | Jingyang | A | Healthy pig | ST10 | 256 | 64 | 1 | 8 | 64 | 2 | <.0.03 | 16 | 32 | 32 | 256 | 32 | 4 | 16 | <.0.03 | TEM-1, CTX-M-9, CTX-M-123 | *qnrS*, *aac(6')-Ib-cr* | *estA*, F17 |
| FP170743 | Fuping | A | Diarrheal pig | ST10 | 512 | 64 | 8 | 64 | 8 | 8 | <.0.03 | 8 | 4 | 64 | 128 | 1 | 1 | 128 | 0.03 | TEM-1, CTX-M-14, CTX-M-123 | *qnrS*, *qnrA* | *estB*, F18 |
| FP170756 | Fuping | A | Diarrheal pig | ST10 | >512 | 128 | 16 | 128 | 64 | 8 | 0.03 | 16 | 8 | 16 | 64 | 32 | 16 | 128 | <.0.03 | TEM-1, CTX-M-15 | *qnrS*, *qnrA* | *astA*, F5 |
| MX161024 | Meixian | A | Diarrheal pig | ST10 | 256 | 64 | 16 | 16 | 32 | 0.25 | <.0.03 | 32 | 16 | 64 | 256 | 16 | 32 | 128 | <.0.03 | TEM-1, CTX-M-15 | *qnrA* | *astA*, F4 |
| HX160912 | Huxian | A | Healthy pig | ST167 | 256 | 64 | 1 | 64 | 8 | 0.125 | <.0.03 | 8 | 2 | 4 | 2 | 2 | 4 | 128 | <.0.03 | TEM-1, CTX-M-64 | *qnrS*, *qnrA* | *estA*, F17 |
| JY160509 | Jingyang | A | Diarrheal pig | ST175 | 128 | 8 | 0.5 | 128 | 16 | 4 | <.0.03 | 16 | 16 | 2 | 128 | 32 | 4 | 128 | <.0.03 | TEM-1,CTX-M-14 | *qnrA* | *estA*, F4 |
| FP160935 | Fuping | A | Healthy pig | ST2715 | 256 | 64 | 1 | 8 | 32 | 1 | <.0.03 | 1 | 0.5 | 2 | 128 | 2 | 4 | 128 | <.0.03 | SHV-12 | - | - |
| JY170618 | Jingyang | A | Diarrheal pig | ST5236 | 256 | 128 | 0.5 | 64 | 64 | 0.5 | <.0.03 | 2 | 1 | 64 | 256 | 32 | 16 | 16 | <.0.03 | TEM-1, CTX-M-14 | *qnrA* | *estA*, F18 |
| HX160826 | Huxian | B1 | Diarrheal pig | ST75 | >512 | 8 | 2 | 4 | 64 | 8 | <.0.03 | 8 | 2 | 16 | 256 | 0.25 | 0.25 | 8 | <.0.03 | TEM-1, CTX-M-14 | *qnrS* | *estA*, F17 |
| ZZ160931 | Zhouzhi | B1 | Healthy pig | ST155 | 256 | 64 | 0.5 | 64 | 32 | 0.125 | 0.03 | 1 | 0.25 | 1 | 128 | 16 | 32 | 128 | <.0.03 | TEM-1, CTX-M-64 | _ | _ |
| ZZ170521 | Zhouzhi | B1 | Diarrheal pig | ST183 | >512 | 64 | 0.25 | 128 | 64 | 0.063 | <.0.03 | 16 | 2 | 2 | 256 | 0.5 | 0.5 | 128 | <.0.03 | TEM-1, CTX-M-14 | *qnrA* | *estA*, *astA*, F6 |
| FP170723 | Fuping | B1 | Healthy pig | ST302 | 256 | 4 | 0.25 | 8 | 64 | 0.25 | <.0.03 | 16 | 2 | 16 | 128 | 0.5 | 1 | 16 | <.0.03 | CTX-M-123 | _ | _ |
| JY170327 | Jingyang | B1 | Diarrheal pig | ST355 | 256 | 16 | 1 | 16 | 128 | 0.5 | <.0.03 | 32 | 0.5 | 0.25 | 256 | 0.25 | 1 | 128 | <.0.03 | TEM-1, CTX-M-14 | *qnrS* | *eae* |
| FF170327 | Fufeng | B1 | Healthy pig | ST443 | 256 | 64 | 0.125 | 32 | 64 | 0.063 | <.0.03 | 0.5 | 0.125 | 1 | 2 | 0.125 | 0.063 | 4 | <.0.03 | CTX-M-14 | *qnrS*, *aac(6')-Ib-cr* | *astA* |
| HX161021 | Huxian | B2 | Diarrheal pig | ST29 | >512 | 128 | 16 | 128 | 64 | 0.063 | 0.03 | 32 | 16 | 32 | 64 | 32 | 16 | 128 | 0.03 | CTX-M-14, CTX-M-123 | *qnrB*, *aac(6')-Ib-cr* | *estA*, F4 |
| FF170425 | Fufeng | B2 | Diarrheal pig | ST95 | 256 | 64 | 32 | 64 | 64 | 4 | 0.063 | 16 | 16 | 64 | 128 | 16 | 32 | 128 | <.0.03 | TEM-1, CTX-M-15, CTX-M-64 | _ | *astA*, F18 |
| MX160918 | Meixian | B2 | Diarrheal pig | ST104 | >512 | 128 | 16 | 64 | 4 | 8 | <.0.03 | 16 | 8 | 32 | 128 | 16 | 32 | 128 | <.0.03 | TEM-1, CTX-M-64 | *qnrS*, *aac(6')-Ib-cr* | *astA*, *estA*, F4 |
| JY160522 | Jingyang | B2 | Diarrheal pig | ST104 | >512 | 256 | 8 | 128 | 64 | 1 | 0.03 | 32 | 32 | 64 | >512 | 64 | 64 | 128 | <.0.03 | TEM-1, CTX-M-64 | *qnrS*, *aac(6')-Ib-cr* | *estA*, F4, F18 |
| HX160809 | Huxian | B2 | Diarrheal pig | ST127 | 512 | 64 | 32 | 64 | 64 | 4 | 0.03 | 32 | 16 | 32 | 256 | 128 | 64 | 128 | <.0.03 | TEM-1, CTX-M-123 | *qnrS* | *estB*, F6 |
| FP170708 | Fuping | B2 | Diarrheal pig | ST127 | >512 | 256 | 8 | 64 | 8 | 16 | <.0.03 | 128 | 64 | 128 | 512 | 64 | 32 | 256 | 0.03 | CTX-M-123 | _ | *estB*, F5 |
| JY160503 | Jingyang | B2 | Diarrheal pig | ST131 | 512 | 32 | 64 | 32 | 64 | 32 | 8 | 128 | 128 | 64 | 128 | 32 | 32 | 128 | 0.063 | CTX-M-9, CTX-M-14, CTX-M-64, KPC-2, OXA-48 | *qnrS*, *qnrB*, *qnrD*, *aac(6')-Ib-cr*, *qepA* | *elt*, *estA*, F6 |
| MX150822 | Meixian | B2 | Diarrheal pig | ST278 | 256 | 64 | 8 | 64 | 16 | 8 | <.0.03 | 32 | 32 | 32 | 256 | 32 | 32 | 128 | <.0.03 | TEM-1, CTX-M-14 | *qnrS* | *astA*, *estB*, F4 |
| FF170416 | Fufeng | B2 | Diarrheal pig | ST355 | >512 | 128 | 32 | 256 | 128 | 8 | <.0.03 | 128 | 64 | 128 | 128 | 128 | 32 | 256 | <.0.03 | SHV-2, CTX-M-64 | *qnrB* | *estA*, F4 |
| FF170325 | Fufeng | B2 | Diarrheal pig | ST372 | >512 | 64 | 16 | 64 | 128 | 4 | 0.063 | 32 | 16 | 32 | 64 | 16 | 16 | 128 | <.0.03 | TEM-1, CTX-M-14, CTX-M-15, CTX-M-64 | *qnrS*, *aac(6')-Ib-cr* | *estB*, F4, F18 |
| FF170316 | Fufeng | B2 | Diarrheal pig | ST372 | 512 | 128 | 32 | 64 | 64 | 0.5 | <.0.03 | 64 | 64 | 128 | 512 | 16 | 32 | 256 | <.0.03 | CTX-M-14, CTX-M-15, CTX-M-123 | *qnrS*, *aac(6')-Ib-cr* | *elt*, *estA*, F4 |
| MX150923 | Meixian | C | Diarrheal pig | ST23 | >512 | 128 | 2 | 64 | 64 | 4 | 0.063 | 32 | 2 | 16 | 2 | 16 | 32 | 128 | <.0.03 | TEM-1, CTX-M-14 | *qnrS*, *qnrA* | *estA*, *estB,* F18 |
| MX150814 | Meixian | C | Diarrheal pig | ST23 | 512 | 64 | 8 | 128 | 32 | 1 | <.0.03 | 32 | 16 | 2 | 256 | 16 | 16 | 256 | 0.03 | TEM-1, CTX-M-14 | *qnrB*, *aac(6')-Ib-cr* | *elt*, F4 |
| ZZ160815 | Zhouzhi | C | Diarrheal pig | ST23 | 512 | 64 | 16 | 64 | 64 | 8 | <.0.03 | 32 | 32 | 64 | 128 | 16 | 1 | 256 | <.0.03 | TEM-1, CTX-M-14 | *qnrS*, | *estA*, F18 |
| JY160518 | Jingyang | D | Diarrheal pig | ST38 | >512 | 128 | 16 | 128 | 32 | 8 | 8 | 64 | 64 | 128 | >512 | 64 | 32 | 256 | <.0.03 | TEM-1, CTX-M-15, OXA-48 | *qnrS*, *qnrA* | *elt, estB*, F4 |
| HX160976 | Huxian | D | Diarrheal pig | ST38 | >512 | 128 | 8 | 64 | 64 | 4 | <.0.03 | 16 | 16 | 256 | 256 | 16 | 16 | 128 | 0.063 | CTX-M-9, CTX-M-14 | *qnrA* | *elt*, *estB*, F41 |
| HX160944 | Huxian | D | Diarrheal pig | ST38 | 512 | 128 | 8 | 128 | 128 | 8 | <.0.03 | 128 | 64 | 64 | >512 | 64 | 32 | 256 | 0.03 | CTX-M-9,CTX-M-14, CTX-M-64 | *qnrB* | *elt*, *astA*, F4 |
| HX161006 | Tianshui | D | Diarrheal pig | ST69 | >512 | 128 | 32 | 64 | 64 | 32 | <.0.03 | 32 | 16 | 128 | 128 | 16 | 16 | 128 | <.0.03 | TEM-1, CTX-M-14 | *qnrS*, *qnrA* | *astA*, F5 |
| MX150820 | Meixian | D | Diarrheal pig | ST405 | 256 | 32 | 64 | 16 | 2 | 32 | 4 | 256 | 128 | 64 | 64 | 64 | 16 | 128 | 0.25 | SHV-12, CTX-M-14, CTX-M-15, NDM-1 | *qnrB*, *aac(6')-Ib-cr* | *elt*, *estA*, F4 |
| LZ161015 | Lanzhou | D | Diarrheal pig | ST405 | 512 | 128 | 16 | 128 | 32 | 1 | <.0.03 | 128 | 64 | 128 | 512 | 64 | 16 | 256 | 0.063 | SHV-12, CTX-M-15 | *qnrS*, *qnrB* | *estB*, F4 |
| JC160611 | Jingchuan | D | Diarrheal pig | ST405 | 512 | 64 | 8 | 64 | 128 | 2 | 1 | 128 | 64 | 256 | 256 | 64 | 32 | 256 | <.0.03 | TEM-1, CTX-M-9, KPC-2 | *qnrS*, *qnrB* | *estB*, F4 |
| JY160512 | Jingyang | D | Diarrheal pig | ST405 | 256 | 32 | 64 | 32 | 128 | 8 | 0.03 | 64 | 128 | 128 | 64 | 32 | 32 | 128 | 4 | TEM-1, CTX-M-9, CTX-M-14, CTX-M-15 | *qnrA*, *qnrD*, *oqxAB* | *elt*, *estA*, F4 |
| FP170711 | Fuping | D | Diarrheal pig | ST405 | 256 | 64 | 64 | 64 | 256 | 32 | 2 | 128 | 64 | 64 | 64 | 64 | 16 | 256 | 0.125 | TEM-1, SHV-12, CTX-M-15, OXA-48 | *aac(6')-Ib-cr, oqxAB* | *elt*, *estB,* F4 |
| HX170832 | Huxian | D | Diarrheal pig | ST405 | >512 | 64 | 8 | 64 | 128 | 16 | 2 | 32 | 32 | 256 | 256 | 32 | 16 | 128 | <.0.03 | TEM-1, SHV-12, CTX-M-15, OXA-48 | *aac(6')-Ib-cr* | *elt*, *estB*, F4 |
| SY160832 | Shanyang | D | Diarrheal pig | ST648 | 256 | 64 | 8 | 128 | 32 | 32 | <.0.03 | 128 | 128 | 16 | >512 | 16 | 32 | 256 | <.0.03 | SHV-2, CTX-M-9, CTX-M-14, CTX-M-123 | *qnrA*, *aac(6')-Ib-cr* | *estB*, *astA*, F17 |
| ZZ160908 | Zhouzhi | D | Diarrheal pig | ST648 | 256 | 64 | 128 | 128 | 1 | 64 | 0.063 | 64 | 128 | 64 | 128 | 32 | 16 | 128 | 0.125 | TEM-1, CTX-M-9, CTX-M-14, CTX-M-15 | *qnrA* | *estB*, *astA*, F41 |
| ZZ160917 | Zhouzhi | D | Diarrheal pig | ST648 | >512 | 128 | 8 | 256 | 128 | 4 | <.0.03 | 64 | 128 | 128 | 256 | 64 | 16 | 128 | <.0.03 | TEM-1, CTX-M-14, CTX-M-15, CTX-M-64 | *qnrS* | *estA*, F18 |
| JY160865 | Jingyang | D | Diarrheal pig | ST648 | 256 | 64 | 16 | 128 | 64 | 8 | 0.03 | 32 | 32 | >512 | >512 | 32 | 8 | 128 | 0.03 | TEM-1, CTX-M-14, CTX-M-15, CTX-M-64 | *qnrS* | *estB*, F18 |
| FP170733 | Puping | E | Diarrheal pig | ST350 | 256 | 64 | 0.125 | 64 | 2 | 4 | <.0.03 | 16 | 0.25 | 2 | 64 | 0.5 | 0.25 | 128 | <.0.03 | CTX-M-9 | *qnrS* | *eae* |

AMP, ampicillin; AMC, amoxicillin-clavulanic acid; EFT, ceftiofur; CTX, cefotaxime; CAZ, ceftazidime; CEX, ceftriaxone; MEM, meropenem;

ENR, enrofloxacin; CIP, ciprofloxacin; FFC, florfenicol, OTC, oxytetracycline; GEN, gentamicin; AMK, amikacin; SXT, sulfamethoxazole-trimethoprim; CLT, colistin.
